# Directed Fusion of Mesenchymal Stem Cells with Cardiomyocytes via VSV-G Facilitates Stem Cell Programming

**DOI:** 10.1155/2012/414038

**Published:** 2012-05-30

**Authors:** Nicholas A. Kouris, Jeremy A. Schaefer, Masato Hatta, Brian T. Freeman, Timothy J. Kamp, Yoshihiro Kawaoka, Brenda M. Ogle

**Affiliations:** ^1^Department of Biomedical Engineering, University of Wisconsin at Madison, Madison, WI 53706, USA; ^2^Department of Pathobiological Sciences, University of Wisconsin at Madison, Madison, WI 53711, USA; ^3^Department of Medicine, University of Wisconsin at Madison, Madison, WI 53706, USA; ^4^The Laboratory for Optical and Computational Instrumentation, University of Wisconsin at Madison, Madison, WI 53706, USA; ^5^The Material Sciences Program, University of Wisconsin at Madison, Madison, WI 53706, USA

## Abstract

Mesenchymal stem cells (MSCs) spontaneously fuse with somatic cells *in vivo*, albeit rarely, and the fusion products are capable of tissue-specific function (mature trait) or proliferation (immature trait), depending on the microenvironment. That stem cells can be programmed, or somatic cells reprogrammed, in this fashion suggests that stem cell fusion holds promise as a therapeutic approach for the repair of damaged tissues, especially tissues not readily capable of functional regeneration, such as the myocardium. In an attempt to increase the frequency of stem cell fusion and, in so doing, increase the potential for cardiac tissue repair, we expressed the fusogen of the vesicular stomatitis virus (VSV-G) in human MSCs. We found VSV-G expressing MSCs (vMSCs) fused with cardiomyocytes (CMs) and these fusion products adopted a CM-like phenotype and morphology *in vitro*. *In vivo*, vMSCs delivered to damaged mouse myocardium via a collagen patch were able to home to the myocardium and fuse to cells within the infarct and peri-infarct region of the myocardium. This study provides a basis for the investigation of the biological impact of fusion of stem cells with CMs *in vivo* and illustrates how viral fusion proteins might better enable such studies.

## 1. Introduction

Mesenchymal stem cells (MSCs) show promise for therapeutic recovery of function of damaged myocardium [1–5]. MSCs home to injured tissues [6, 7] and contribute to the structure or functional recovery of the myocardium via (1) secretion of paracrine factors that can inhibit immune responses [8] and/or facilitate angiogenesis [7, 9, 10], (2) transdifferentiation/metaplasia [11, 12], and (3) nuclear reprogramming through fusion with resident cardiomyocytes (CMs) [13]. The latter has been largely dismissed since the frequency at which fusion is detected is low relative to the number of transplanted MSCs. However, recent studies by us [14] and others [15–17] suggest that despite the low frequency cell fusion still may exert a dramatic impact on stem cell programming or reprogramming in the heart.

Cell fate determination was once thought to be unidirectional [18], that is, as progenitor cells differentiate there is a progressive and permanent inactivation of specific genes that allow for their potency. However, technological advances suggest this is not strictly the case. Pioneering experiments of nuclear reprogramming utilized cell fusion to demonstrate that cytoplasmic elements of one fusion partner can impact nuclear transcription factors of the other fusion partner, inducing programming or reprogramming [19–21]. Later studies pinpointed specific transcription factors that, when activated exogenously, can fully reprogram somatic cells to an embryonic-like state [22–26]. Though successful reprogramming has been realized with this tailored *in vitro* approach, programming may require greater temporal control. Spontaneous physiologic cell-cell fusion is a temporally and spatially regulated process essential for programming or differentiation of certain cell types [27, 28]. Thus cell fusion may also confer a regulated transfer of transcriptional control necessary to drive stem cell or progenitor cell differentiation for repair of tissues in mature animals.

Cell-cell fusion occurs when the plasma membranes of neighboring cells fuse to form a multinucleated cell. To fuse, lipid bilayers of cell membranes must come into very close contact, in the range of several angstroms. To achieve this degree of close proximity, the two surfaces must become at least partially dehydrated as water bound to the membrane enhances polar repulsion of membranes. Next, one or both bilayers must be destabilized in some way, inducing a localized rearrangement of the bilayers. If both bilayers are destabilized, an aqueous bridge is formed and the cytoplasmic contents of both cells mix.

Destabilization of membranes can occur as the result of physical stress (e.g., electrofusion) or chemical interference (e.g., polyethylene glycol). Electrofusion utilizes short pulses of electricity to mechanically disrupt the lipid bilayer of a cell to form pores and if two disrupted membranes come into contact, cell fusion may occur [29]. Unfortunately, this process is toxic and the cells must be in contact with one another at the time the electric field is administered. Laser trapping prior to electrofusion has been used to more effectively position fusion partners, however the process is low throughput and cytotoxic [30, 31]. A less toxic, but also less effective and less reproducible approach uses polyethylene glycol (PEG) [32, 33]. The exact mechanism of PEG-induced fusion is unknown but is theorized to be due to either local dehydration leading to unfavorable molecular packing of the bilayer or to dehydration of the “water shell” near the lipid bilayer, causing the water molecules between cells to be displaced, thereby forcing the two membranes together and subsequently fusing the cells [34]. This technique has proven useful, but fusion only occurs during the time of administration of PEG, thus cell delivery with PEG would induce fusion immediately and nonselectively. A mechanism that would better regulate fusion either to specific cells or specific regions within tissues is necessary to study fusion *in vivo*.

In nature, destabilization of cell membranes and subsequent membrane fusion utilizes the activation of specific integral membrane proteins, termed fusogens. The primary source of information about fusogen architecture, receptor binding, and activation are from viruses. The most extensively characterized fusogens are influenza hemagglutinin (HA) and human immunodeficiency virus type 1 envelope protein (HIV-1 Env). Both fusion peptides are hydrophobic and require proteolytic cleavage, but HA is activated under acidic pH during endocytosis, while HIV-1 Env fuses at neutral pH (reviewed in [35–37]). Less well-studied are the fusogens required for eukaryotic cell fusion such as the fusion of osteoclasts, myoblasts, and trophoblasts. The greatest challenge has been establishing which proteins are true fusogens and which proteins facilitate fusion by placing cells in close proximity. Many putative fusogens have been shown to be supporting proteins (i.e., essential for adhesion or migration). The identification of true fusogens is so difficult that groups have proposed ranking schemes to clarify the nature and function of these proteins [28]. Because putative fusogens for spontaneous stem cell fusion have not been identified, developing alternative strategies to direct stem cell fusion could augment our understanding of the biological impact of such fusion.

Here we utilize viral machinery from vesicular stomatitis virus (the glycoprotein, VSV-G), of the *Rhabdoviridae* family, to induce heterotypic fusion between human MSCs and mouse CMs *in vitro *and in an *in vivo* mouse model of myocardial infarction. Following MSC-CM fusion, we tracked the phenotype and morphology of fusion products for one week *in vitro *and 3 weeks *in vivo*. VSV-G was selected because it does not require proteolytic cleavage, is the sole mediator of receptor binding and fusion, and is pH dependent [38, 39]. In particular, VSV-G does not require facilitating proteins to either dock to the host membrane prior to fusion, or enzymes to prompt the activation of the fusogen. Furthermore, the pH dependence of VSV-G is advantageous as the local heart pH after acute ischemic injury [40–42] is within the acidic range needed to initiate a conformational change in VSV-G [38, 39]. In this way, selective activation of VSV-G on transfected MSCs (vMSCs) at the site of myocardial injury should induce local fusion *in situ*, thereby increasing donor cell engraftment and integration within the tissue and potentially facilitate cardiac differentiation.

## 2. Materials and Methods

### 2.1. Cell Culture

MSCs derived from human embryonic stem cells (MSCs from WA-09, a gift of Dr. Peiman Hematti) and HL-1 cardiomyocytes (a gift of Dr. William Claycomb) were expanded and cultured as previously described [43, 44]. Briefly, MSCs were cultured on a 0.1% gelatin (Sigma-Aldrich, St. Louis, MO, USA) pretreated flask containing *α*-minimum essential medium- (MEM-) complete. Alpha-MEM-complete consisted of *α*-MEM (Invitrogen, Carlsbad CA, USA), 10% fetal bovine serum (Hyclone, Logan UT), 0.1 mM nonessential amino acids (Invitrogen), and 2 mM *L*-glutamine (Invitrogen). MSC cultures were allowed to grow to 60–70% confluency and were replated at a concentration of 1,500 cells/cm^2^. CMs were cultured on fibronectin/gelatin (1.25 mg fibronectin/100 mL 0.02% gelatin) (Sigma-Aldrich) pretreated flasks containing Claycomb-complete. Claycomb-complete medium was comprised of Claycomb medium (SAFC Biosciences, St. Louis, MO, USA), 10% fetal bovine serum qualified for CMs (SAFC Biosciences), 100 U/mL: 100 *μ*g/mL penicillin-streptomycin (Lonza, Walkersville, MD, USA), 0.1 mM norepinephrine (Sigma-Aldrich), and 2 mM *L*-glutamine (Invitrogen). CMs were passaged at 100% confluence and split 1 : 2. Experiments were performed using passages 7–10 and 60–110 for MSCs and CMs, respectively. All cultures were maintained at 37°C in 5% CO_2_.

### 2.2. Transfection and Analysis

MSCs were transfected with a pCVSV-G-1 plasmid [45] that encodes VSV-G under a CAG promoter using the Neon Transfection System (Invitrogen), according to manufacturer's protocol. Briefly, 5 × 10^5^ cells were transfected with 2 *μ*g of plasmid with one 1,300 V pulse for 20 msec and plated in 6-well plates. To determine transfection efficiency, electroporated cells were cultured for 24 h and immunocytochemistry (ICC) was performed to detect VSV-G protein expression. Briefly, cells were washed with two rinses and two incubations of 1X PBS. Cell fixation was performed with 4% PFA, followed by another set of washes, and probed with the 1 : 50 dilution of FITC-conjugated anti-VSV-G antibody (GeneTex, San Antonio TX, USA) in 3% BSA for 60 min. Cultures were washed a final time and mounted in DABCO/DAPI mounting medium (2.5% 1,4-diazabicyclo[2.2.2]octane (Sigma-Aldrich), 50% glycerol (Fisher Scientific, Forest Lawn, NJ, USA), and 0.005% 4′,6-diamidino-2-phenylindole (Sigma-Aldrich) in PBS). The transfection efficiency was calculated as the number of cells positive for VSV-G (green) divided by the total number of cells. To determine if the cell dissociation reagent altered VSV-G expression, duplicate VSV-G transfected MSC (vMSC) cultures were harvested with 0.25% trypsin (Mediatech, Inc. Manassas, VA, USA) or 1X Accutase (Innovative Cell Technologies, Inc. San Diego, CA, USA) containing 0.5 mM EDTA, inactivated with culture medium, washed with 1X PBS, probed with the anti-VSV-G antibody (as above), and washed a final time with 1X PBS. vMSCs were analyzed via FACSCalibur (BD Biosciences San Jose, CA, USA) at the University of Wisconsin Carbone Cancer Center Flow Cytometry Facility (UWCCC).

### 2.3. Flow Cytometry Analysis

VSV-G expression level per cell was determined using the Quantum MESF kit (Molecules of Equivalent Soluble Fluorochrome, Bangs Laboratories, Inc. Fishers, IN) and FACSCalibur cytometer (BD Biosciences). The Quantum MESF kit consists of 5 populations of microspheres with increasing surface-labeled fluorochrome, which have been standardized to specific concentrations of pure fluorophore per microsphere. Each population was analyzed via flow cytometry and a standard curve was generated by plotting population (i.e., concentration of fluorophore per microsphere) versus intensity. QuickCal software was used to verify the linearity of the standard curve. Next, vMSCs and corresponding control populations were labeled with an anti-VSV-G-FITC antibody and analyzed via flow cytometry. Using the standard curve and the measured intensity value for vMSC populations and corresponding controls, the number of fluorophores per cell was determined. This value was divided by the average number of fluorophores (4.2) that bind to a single antigen to determine the number of proteins expressed per cell. Ten thousand cells and three replicates were analyzed per population. Populations included vMSCs with anti-VSV-G antibody, MSCs with anti-VSV-G antibody and vMSCs without antibody.

### 2.4. Cell Fusion Induction

To determine if vMSCs fuse more readily with cardiomyocytes than untreated MSCs, vMSCs and MSC controls were cocultured with CMs and analyzed for incidence of fusion. To distinguish cell types in cocultures, MSCs and CMs were stained with 1 *μ*m CellTracker Green CMFDA and 20 *μ*m Red CMTPX (Molecular Probes Eugene, OR, USA), respectively, according to the manufacturer's protocol. Following labeling, 5 × 10^5^ CMs were plated and cultured for 4 h followed by the addition of 1.5 × 10^5^ MSCs or vMSCs per well in 6-well plates (BD Biosciences). After 14 h of coculture, suspensions were washed with 1X PBS and then bathed for 2 min in fusion media [46] of varying pH (i.e., pH 5.5, 6.5, or 7.5 that correspond to active and inactive forms of the VSV-G fusion protein) adjusted with HCl. For long-term characterization of fusion products, medium was changed 1 day and 4 days after coculture.

### 2.5. Quantification of Fusion Products

Cocultures of CMs with MSCs or vMSCs were maintained in culture medium for 4 h after incubation with fusion medium, followed by imaging and flow cytometry. Images were acquired with a 20X UPlanFluor objective (NA = 0.5), FITC and Texas Red filters, on an IX71 inverted deconvolution fluorescence microscope (Olympus Center Valley, PA, USA) and analyzed with Slidebook software (Intelligent Imaging Innovations Denver, CO, USA) and ImageJ (Fiji; open source software, http://pacific.mpi-cbg.de/wiki/index.php/Fiji). Images were normalized using unstained controls. Cells were analyzed at the UWCCC Flow Cytometry Facility on a FACSCalibur flow cytometer (BD Biosciences). Events were live/dead gated with forward scatter and side scatter plots. Fusion products were quantified by gating the region positive for FL1 and FL2 channels, corresponding to CellTracker Green CMFDA and Red CMTPX, respectively.

### 2.6. Optical Analysis of Cell and Fusion Product Phenotype

MSCs or CMs in monolayer were stained for proteins characteristic of MSCs (CD73, CD90, and CD105), as well as proteins characteristic of CMs (sarcomeric myosin (MF20)). Cell cultures were fixed with 4% paraformaldehyde for 10 min, followed by two washes with phosphate buffered saline (Fisher Scientific). Cells were probed with goat anti-CD73 (V-20, Santa Cruz Biotech, Santa Cruz, CA, USA), rabbit anti-CD90 (RB3970, Abgent, San Diego, CA, USA), goat anti-CD105 (GKY02, R&D Systems, Minneapolis, MN, USA), and mouse anti-MF20 (IgG2b, Developmental Studies Hybridoma Bank, Iowa City, IA) diluted 1 : 25, 1 : 50, 1 : 50, and no dilution, respectively, in diluting buffer (5% BSA (Fisher Scientific), 0.02% NaN3- (Acros Organics) in phosphate buffered saline (Fisher Scientific)) and incubated for 30 min at room temperature or overnight at 4°C, followed by incubation with fluorescent secondary antibodies: donkey anti-goat Alexa Fluor (AF488, Invitrogen), goat anti-rabbit Alexa Fluor (AF647, Invitrogen), and donkey anti-mouse (AF546, Invitrogen) at a 1 : 200 dilution in preadsorption solution (90% diluting buffer, 5% human serum (Pelfreez, Brown Deer, WI, USA), and 5% mouse serum (Equitech-Bio, Inc, Kerrville, TX, USA)) for 45 min at room temperature. Samples were counterstained with DABCO/DAPI mounting solution. Fluorescence emission was detected on an IX71 inverted deconvolution fluorescence microscope (Olympus). Images were acquired with a 20X UPlanFluor objective (NA = 0.5), and analyzed using Slidebook software (Intelligent Imaging Innovations Denver, CO, USA) and with ImageJ (Fiji; open source software, http://pacific.mpi-cbg.de/wiki/index.php/Fiji). Images were thresholded to a secondary antibody only control. Validation of method is shown in Supplementary Figure 1A available online at doi:10.1155/2012/414038. 

MSC-CM or vMSC-CM cocultures were probed with antibodies against CM marker (MF20) and MSC marker (CD105) to evaluate the morphology of fusion products and the phenotype of cells within coculture. Positive events for fusion were calculated as the percentage of CD105 and MF20 positive cells containing a nucleus divided by total number of nuclei obtained from analysis of at least eight optical fields per sample. Fields (3–10 fields) were selected based on cell number (minimum of 3 cells) and position within the wells (center of wells) *n* = 2.

### 2.7. Induction of Myocardial Infarction in Mice

Myocardial infarction was induced in C57BL/6 mice (Jackson Laboratory, Bar Harbor, ME, USA) by left coronary artery ligation as previously described [47, 48] and as is routinely performed in the University of Wisconsin Cardiovascular Physiology Core Facility. All animal procedures were performed in accordance with the guidelines of the American Association for Laboratory Animal Science and the University of Wisconsin-Madison Animal Care and Use Committee.

### 2.8. Delivery of MSCs or vMSCs via the TissueMend Matrix to the Murine Myocardium

TissueMend (TEI Biosciences) was prepared and cells were seeded as previously described [48]. Briefly, TissueMend matrices (2 mm × 2 mm × 0.8 mm) were placed in wells of 24-well plates containing *α*-MEM-complete culture medium. Following electroporation, vMSCs were seeded on the TissueMend sections at a concentration 3 times greater than MSCs (cell control due to 30% cell viability after electroporation, yielding 1 × 10^6^ cells/mL. Medium was changed at 24 and 48 h, at which point the TissueMend matrix containing ~2.3 × 10^4^ MSCs, vMSCs, or unseeded (matrix control) was tacked to the myocardium at each corner of the matrix. The matrix was placed such that it was in contact with both the infarct and the peri-infarct regions of the myocardium [48].

### 2.9. Optical Analysis of Heart/Tissue Explants

Murine hearts were harvested three weeks after matrix implantation to assess the occurrence and, if detected, the frequency of *in vivo *fusion. Hearts were bisected longitudinally through the matrix. The tissues were immediately placed into 10% buffered formalin (pH = 7.2; Fisher Scientific) for 24 h followed by 24 h of fresh 10% buffered formalin, and a final 24 h of 70% ethanol. Samples were further processed for paraffin embedding and sectioning as previously described [49]. Fluorescent in situ hybridization (FISH) tissue digestion kit with all human centromere probe (red) and all mouse centromere probe (green) (Kreatech, Amsterdam, the Netherlands) was performed on sections to detect fusion events. Samples were processed by the Cytogenetics Laboratory (WiCell Research Institute, Madison, WI, USA) according to manufacturer's protocol. Briefly, slides with paraffin embedded sections were baked for 4 h at 56°C. Specimens were incubated with pepsin for 70 min for tissue digestion prior to sequential hybridization of the human probe followed by mouse probe. Images were acquired with a 60X UPlanSApo (NA = 1.35 Oil), DAPI, Green, and Orange filters, on an Olympus BX41 Upright Fluorescence Microscope (Olympus Valley, PA, USA), and analyzed with FISHView Version 5.5 software (Applied Spectral Imaging, Vista CA). Fusion events were defined as nuclei with positive staining for both human centromeres (red) and mouse centromeres (green). The frequency of fusion events was reported as the number of fusion events per total nuclei for a given region of the heart tissue: myocardium (myo), myocardial infarct (MI), border region (border), within Tissuemend patch (TM), and in the healthy myocardium (healthy). Five to twelve fields were selected for each location and the number of hearts analyzed were, *n* = 1 for TM only and *n* = 3 for TM + vMSC.

### 2.10. Statistical Analysis

For comparison of VSV-G expression, fusion frequency, and fusion product morphology versus controls, a normal distribution was assumed and one-way analyses of variance and Student's *t*-test were used. Data were analyzed with Microsoft Excel (Microsoft, Redmond, WA, USA).

## 3. Results

### 3.1. Expression of VSV-G in MSCs

**Figure 1:**
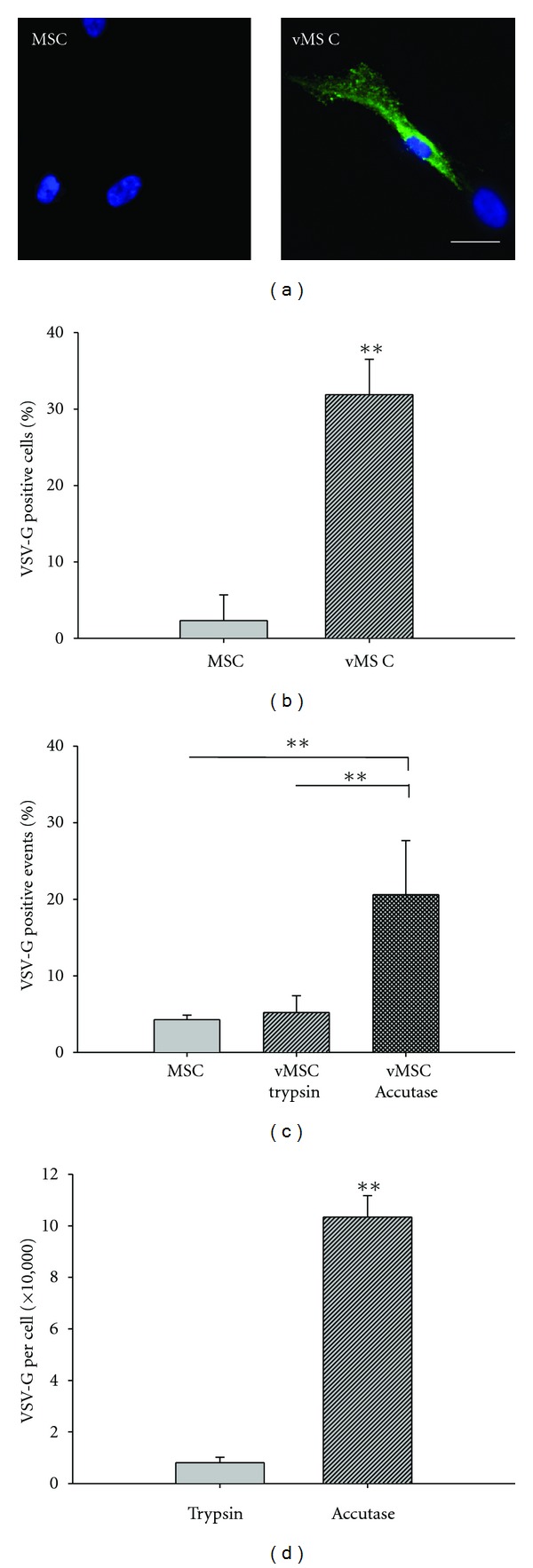
Expression of VSV-G in MSCs. VSV-G expression was analyzed via immunofluorescence using an anti-VSV-G-FITC antibody. (a) Representative image analysis of untransfected MSCs and vMSCs; VSV-G (green); DAPI (blue). Scale bar = 25 *μ*m. (b) Transfection efficiency was defined as the number of VSV-G-positive cells divided by the total number of nuclei and is reported as the mean ± standard deviation. A low level of nonspecific binding was associated with the anti-VSV-G antibody and is reflected in the percentage of positive cells reported in the untransfected population of MSCs (2.3% ± 3.4%). (c) Dissociation reagent impacts VSV-G expression. Trypsin treatment reduces detection of VSV-G expressing cells to that of untransfected MSCs. Accutase treatment retains a significantly greater number of cells expressing VSV-G than treatment with trypsin; **P < 0.005. (d) Dissociation reagent impacts the number of VSV-G proteins per cell. The number of VSV-G proteins expressed per cell is significantly reduced with trypsin treatment as compared with Accutase treatment, which was quantified utilizing Quantum Simply Cellular standards; **P < 0.005.

MSCs were induced to express VSV-G via transfection by electroporation. Low transfection efficiency would limit the ability of VSV-G to promote fusion and so VSV-G expression on MSCs was determined following electroporation. Twenty-four hours after transfection control MSCs and MSCs transfected with VSV-G (vMSCs) were probed with an anti-VSV-G antibody conjugated to fluorescein isothiocyanate (FITC) and visualized with fluorescence microscopy to determine the percentage of cells expressing VSV-G. The average transfection efficiency was 32% ± 5% (*n* = at least 6 optical fields per sample per trial for 3 trials, Figure 1(a)). Since vMSCs will be harvested for *in vivo *studies, we also assessed VSV-G expression via flow cytometry after removal from culture plates with trypsin. We found expression of VSV-G plummeted to 5% ± 2% (*n* = 1 replicate per sample per trial for 3 trials, Figure 1(b)). This is perhaps not surprising as others have reported decreased stability of VSV-G with trypsin treatment [50, 51]. Trypsin is a serine protease that cleaves carboxyl groups on the cell surface to remove cells from a culture surface. VSV-G is a cell surface protein that would be exposed to the dissociation reagent [52]. The disruption to VSV-G by trypsin was corroborated by evaluating the number of VSV-G proteins per cell. The administration of trypsin significantly reduced the number of VSV-G proteins on the cell surface of vMSCs (Figure 1(d)). Thus we replaced trypsin with Accutase, a mixture of proteases and collagenases that has been shown to improve cell viability compared to trypsin [53]. With Accutase treatment, the average number of cells expressing VSV-G after cell harvest was 21% ± 7%, a significant improvement over treatment with trypsin and at a level high enough to discern whether expression of VSV-G can impact MSC-CM fusion (*n* = 1 replicate per sample per trial for 3 trials).

Fusogens such as VSV-G are most potent on the cell surface when there are adequate amounts of protein to facilitate fusion, but a low enough amount to avoid immune responses. Thus VSV-G protein expression per cell was determined using a Quantum MESF kit (Bangs Laboratories, Inc.), on the FACSCalibur. Using this method, the average number of VSV-G proteins per cell was 8 × 10^4^ ± 2 × 10^4^ with trypsin and 1 × 10^6^ ± 8 × 10^3^ with Accutase (*n* = 1 replicate per sample per trial for 3 trials, Figure 1(c)). Thus, all further experiments were performed using Accutase as the dissociation reagent to prevent VSV-G cleavage.

### 3.2. VSV-G Mediates Stem Cell Fusion

**Figure 2:**
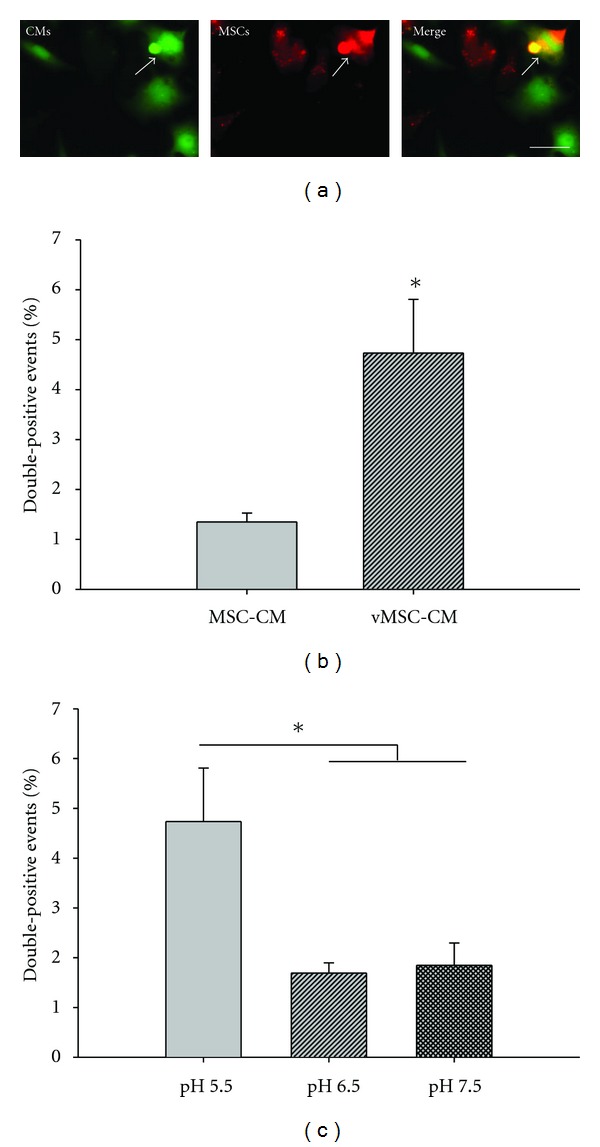
VSV-G facilitates vMSC-CM fusion. Four hours after pH-induced fusion, samples were harvested and analyzed using fluorescence deconvolution microscopy and flow cytometry for events displaying both CellTracker fluorescent probes. (a) Representative vMSC-CM fusion event (white arrow). CMs were labeled with CellTracker green and vMSCs were labeled with CellTracker red. Scale bar = 50 *μ*m. (b) Effect of VSV-G on vMSC-CM fusion at pH = 5.5. Fusion events were increased 3.5-fold with VSV-G at low pH. (c) Effect of pH on VSV-G-mediated MSC-CM fusion. vMSC-CM fusion was inhibited by pH = 6.5 and 7.5 (inactive form of VSV-G) *P < 0.05.

To determine whether MSCs expressing VSV-G are better equipped to fuse with CMs than unmanipulated counterparts, vMSCs or MSCs were cocultured with CMs. To distinguish cell types in cocultures, MSCs and vMSCs were stained with Red CellTracker, while CMs were stained with Green CellTracker fluorescent probes prior to being combined. Since VSV-G undergoes a conformational change from its inactive form to its active form at pH < 6.2 [54, 55], cocultures were briefly incubated (2 min) with fusion medium of pH = 5.5. Image analysis of vMSC-CM cocultures treated with fusion medium of pH = 5.5 revealed cells with colocalization of green and red fluorescence, indicating fusion events, while MSC-CM cocultures under the same pH condition exhibited limited colocalization (Figure 2(a)). To accurately assess the amount of cell fusion, cocultures were harvested 24 hours after seeding and analyzed via flow cytometry (double positive cells correspond to fusion events). vMSCs treated with acidic medium (pH 5.5) had significantly higher rates of fusion with CMs (4.7% ± 1.1%) than MSCs treated in the same way (i.e., spontaneous fusion, 1.4% ± 0.2%) (*P* < 0.05) (Figure 2(b)). Further, the percentage of fusion products identified in vMSC-CM cocultures exposed to fusion medium of pH = 6.5 or 7.5 (maintaining VSV-G in the inactive form) did not differ from MSC-CM cultures (*n* = 3 replicates per sample per trial for 3 trials, Figure 2(c)).

### 3.3. Impact of Microenvironment on Phenotype of MSC-CM Fusion Products

**Figure 3:**
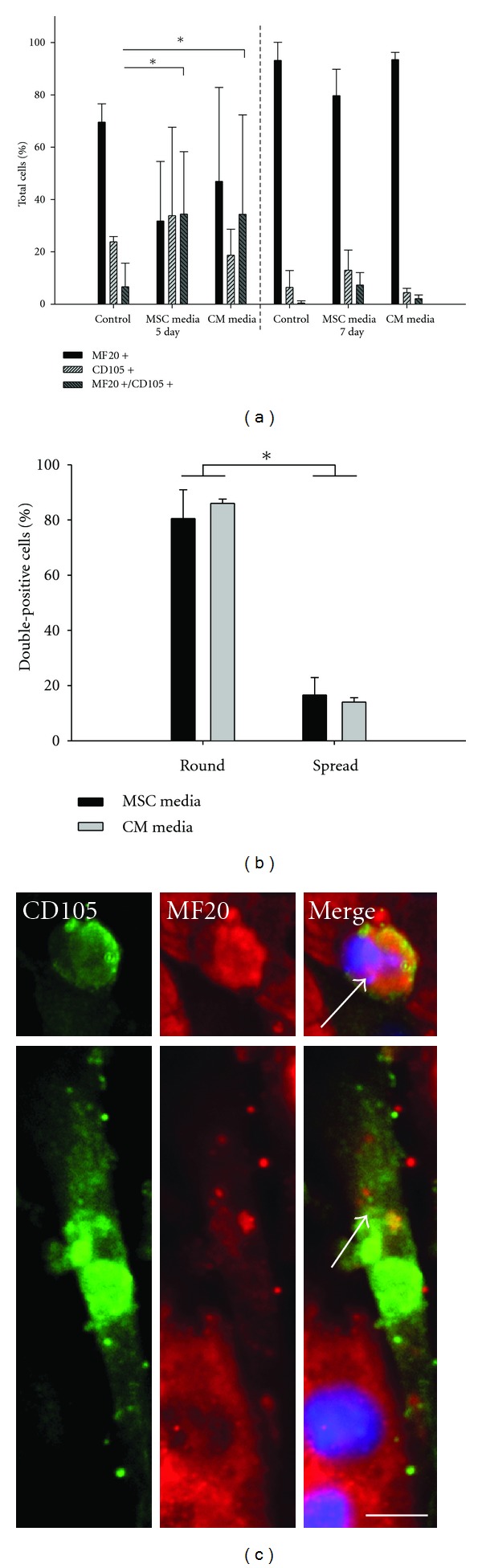
Phenotypic and morphologic characteristics of vMSC-CM fusion products. After pH-induced fusion, fusion products display two distinct morphologies: CM-like (round) and MSC-like (spread). Fusion products were probed for markers indicative of MSC (CD105) and CM (MF20) phenotype. (a) MF20^+^/CD105^+^ cells are significantly increased with vMSCs at day 5 relative to untransfected MSCs, while the culture environment (MSC medium and CM medium) had no effect on the percent of dual positive events in vMSC-CM cocultures at day 5. *P < 0.05. (b) Morphology of MF20^+^/CD105^+^ cells was typically CM-like and culture environment did not alter this tendency. *P < 0.05. (c) Representative morphologies of MF20^+^/CD105^+^ cells. White arrows indicate MF20^+^/CD105^+^ cells. Scale bar = 25 *μ*m.

Many studies have demonstrated that stem cell programming is influenced by the microenvironment [56–58]. To determine whether the phenotypic fate of vMSC-CM fusion products could be regulated by the microenvironment, following treatment with fusion media, we cultured vMSC-CM fusion products under either MSC-specific or CM-specific culture conditions and examined the incidence of fusion and morphology of MSC-CM fusion products. At days 5 and 7 following the induction of cell fusion, cocultures were probed with CM and MSC specific antibodies (anti-MF20 and anti-CD105, respectively, *n* = 1 replicate per sample per trial for 3 trials). At day 5, vMSC-CM cocultures contained a relatively high number of cells that expressed both MF20 and CD105 and the percentage of MF20^+^/CD105^+^ cells relative to the total cell number was significantly greater than that of MSC-CM cocultures for both culture conditions (*P* < 0.005) (Figure 3(a)). Of note, the percentage of MF20^+^/CD105^+^ cells was much higher than the percentage of double positive cells detected using CellTracker dyes and flow cytometry (Figure 2(b)). This could reflect the loss of VSV-G sustained by cell harvest, the different analytical approach (i.e., flow cytometry versus image analysis) and/or the behavior of fusion products between day 1 and day 5 (i.e., proliferation). By day 7, the percentage of MF20^+^/CD105^+^ cells decreased to levels not statistically different from controls for both culture conditions. At the same time, the number of cells expressing MF20 alone increased substantially for both culture conditions. The change in percentage of MF20^+^/CD105^+^ cells from day 5 to day 7 could reflect death of fusion products, or programming of the MSC fusion partner to a cardiomyocyte phenotype or both. If death of fusion products occurred, one would expect unfused CMs and MSCs to proliferate to fill the voids of the culture space. Interestingly, only the CM population increased from day 5 to day 7 and at rates significantly higher than that of control cultures, suggesting at least a portion of fusion products were maintained, and ultimately adopted a cardiomyocyte-like phenotype. This result was observed independent of the culture conditions. Of note, this experimental approach does not exclude the possibility that metaplasia rather than fusion occurred, that is, MSCs differentiate into CMs as a consequence of soluble factors in the coculture medium and maintain (at least transiently) expression of each cell type. However, MF20^+^/CD105^+^ cells were rare in MSC-CM cocultures, suggesting metaplasia alone cannot account for coexpression of MF20^+^/CD105^+^ or subsequent loss of MF20^+^/CD105^+^ cells. In addition, MF20^+^/CD105^+^ cells exhibited two distinct morphologies; some were long and spread, displaying MSC-like morphology (MSC medium = 16.59% ± 6.32%; CM medium = 14.03% ± 1.59%) while the majority (*P* < 0.05) were round and cobblestone-like, indicative of CM-like morphology (MSC medium = 80.49% ± 10.45%; CM medium = 85.97% ± 1.60%) (Figures 3(b) and 3(c), Supplementary Figure 1B). These results further support the possibility that CM nuclear material and cytoplasmic elements direct programming of MSC-CM fusion products independent of culture conditions.

### 3.4. vMSCs Fuse *In Vivo*

**Figure 4:**
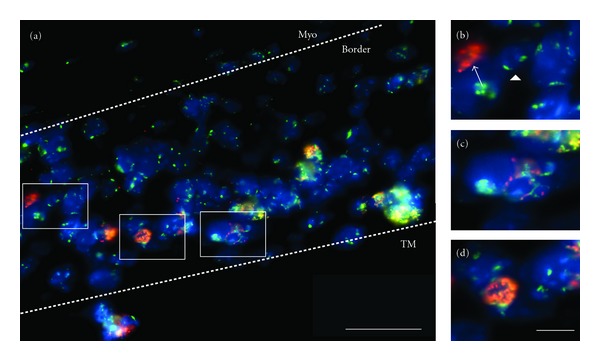
Fusion of VSV-G-expressing human mesenchymal stem cells (donor) with cardiomyocytes (host) *in vivo*. (a) Fluorescence in situ hybridization with species-specific centromeric probes to detect fusion products in the murine myocardium. Human MSCs (red), mouse cells (green), all nuclei (blue), and fusion products (FP; red and green signal in the same nucleus) are found within the border and TM regions, while typically only mouse cells are found in the myocardium, “myo.” Insets display (b) human (arrow) and mouse (arrowhead) cells, (c) representative fusion product, and (d) fusion product undergoing anaphase, indicative of proliferation.

To determine whether MSCs expressing VSV-G could fuse with cardiac cell types *in vivo*, vMSCs were delivered to the damaged myocardium via a TissueMend patch. We have previously demonstrated that MSCs delivered in this way are maintained in the patch and in the tissue between the patch and the myocardium up to 3 weeks after delivery at higher percentages than with conventional delivery modalities [48]. Furthermore, Laflamme et al. have found one of the major factors for cell loss during transplantation is anoikis [59], and thus providing anchorage support to transplanted cells increases viability and retention. In this study, we sought to determine whether VSV-G expressing MSCs (donor) would be able to migrate to the damaged myocardium and fuse with recipient cardiac cell types. Thus, one day following induction of infarction via ligation of the left anterior descending artery, a patch containing vMSCs was applied to the heart in contact with healthy and damaged tissue. Three weeks after cell transplantation, heart excision, and histology were performed on left ventricular tissue as previously reported [48]. Histological sections were probed using FISH for human-specific and mouse-specific centromeres and all nuclei containing both probes were considered fusion products. Human cells were found in the TissueMend patch and in the “border region” (the area between the patch and the myocardium). Donor-host cell fusion was evident in the TissueMend patch, the border zone, and in the infarcted myocardium of hearts receiving TissueMend with vMSCs. No human cells or fusion products were found in the healthy cardiac tissue of hearts receiving TissueMend with vMSCs. In addition, no human cells or fusion products were found in the TissueMend patch, border zone or infarcted myocardium of hearts receiving TissueMend only. In regions of hearts receiving TissueMend with vMSCs and selected for high density of fusion events, the frequency of cell fusion relative to the total number of nuclei in a given region was 22% ± 16%, TissueMend patch (*n* = 3 hearts, 12 fields); 14% ± 10%, border zone (*n* = 3 hearts, 5 fields); 19% ± 10%, infarcted myocardium (*n* = 3 hearts, 8 fields). Though these levels represent the maximum amount of fusion per region, they are substantially higher than those previously reported for spontaneous fusion following MSC transplantation, wherein one fusion event per field or image containing hundreds of nuclei was rare [13, 60, 61] (Figure 4). These results demonstrate that expression of viral fusogen VSV-G can be used to induce fusion of MSCs, and potentially other clinically relevant cell types, to enable study of the biologic and therapeutic impact of cell fusion in the heart.

## 4. Discussion

Fusion of transplanted stem cells with recipient cardiomyocytes has been observed in murine [13, 60] and porcine model systems [49]. But since these first observations, few have sought to unravel the mechanisms that govern stem cell fusion or to study the implications of cell fusion for stem cell programming. Lack of study reflects the overwhelming opinion that cell fusion occurs too infrequently to be of relevance for stem cell programming and, by corollary, for tissue repair. However, this opinion fails to appreciate the possibility that (1) detection methodologies may be insufficient to accurately gauge the contribution of cell fusion following stem cell transplantation and/or (2) that we might be able to control or increase the frequency of cell fusion to more effectively induce programming of stem cells following transplantation. We have begun to explore this second possibility by co-opting the well-described fusion machinery of viruses. We find that mesenchymal stem cells modified to express viral fusogen VSV-G are more apt to fuse with cardiomyocytes in a pH-dependent manner. vMSC-CM fusion products formed in this way are prone to adopt cardiomyocyte phenotype and morphology. In addition, vMSCs delivered to the myocardium of mice following infarction can fuse with resident cardiac cell types at rates much higher than previous reports of spontaneous fusion [13, 61] and are more apt to fuse at the site of infarction than in the healthy myocardium.

Increasing the frequency of MSC-CM cell fusion will aid in the study of cell fusion *in vitro *and may improve the therapeutic benefit of MSCs *in vivo. *One way that the therapeutic benefit may be improved is via induction of programming of MSCs to a cardiomyocyte fate. Differentiation of MSCs into CMs can be initiated *in vitro *via soluble factors including 5-azacytidine [62–64] or with exposure to insoluble factors including laminin [65]. However, functional differentiation of MSCs to cardiomyocytes has only been accomplished to date via cell fusion with mature cardiomyocytes. This result has been demonstrated *in vitro* [66] and *in vivo *wherein MSC-CM fusion products take on a cardiomyocyte morphology, express cardiomyocyte markers, and couple to adjacent cardiomyocytes [60]. Here we find that when MSC-CM fusion is induced with viral fusogens, the CM fusion partner is dominant in that the majority of fusion products (regardless of medium type) adopt a CM-like morphology and maintain expression of MF20 and lose CD105. These data further support the exciting possibility that induction of fusion with viral fusogens could enhance MSC programming to a CM fate *in vivo*. Of note, the CMs utilized here are HL-1 CMs. This cell line was used to enable large-scale and long-term studies. However, the heterogeneity and immortal nature of these cells may account for the seeming dominance of the CM phenotype and future studies will utilize primary fetal cardiomyocytes or induced pluripotent stem cell-derived cardiomyocytes.

Our results suggest that the differentiation of MSCs to a CM fate can be promoted by cell-cell fusion. However, in certain circumstances *in vitro*, MSC-CM fusion products can reenter the cell cycle and proliferate suggesting cell-cell fusion can also promote reprogramming of the CM [67–69]. Proliferation of fusion products may be as advantageous for cardiac tissue repair as differentiation of functional cell types since more cells could be produced to replace lost cells. In addition, recent evidence has demonstrated that MSC-CM fusion includes mitochondrial exchange, which is essential for somatic reprogramming [69]. Understanding cell-cell fusion in conjunction with mitochondrial preservation may provide alternate, simple, and direct mechanisms to rescue cells following ischemia-induced damage. There is evidence indicating that the fusion product's proliferative capacity is regulated by the stem cell while the developmental direction is dictated by the somatic cell [70–72], and the combination of both outcomes presented herein are means to repopulate the myocardium for functional improvement.

While we have utilized vMSCs to both understand and exploit the physiological role of MSC-CM fusion, induction of fusion of another stem cell, progenitor, or even mature cell types may augment our ability to repopulate and repair the damaged myocardium [59, 73–79]. In the case of mature or progenitor cell transplantation, the induction of fusion may be less beneficial from a differentiation standpoint and more beneficial from an engraftment or retention standpoint. One of the primary challenges for stem cell delivery is the ~90% cell loss after transplantation [80–82] that has prompted the development of new methods to deliver and maintain cells in the heart [48, 83, 84]. This is particularly problematic for cardiac therapy as the heart is mechanically active, rapidly flushing cells from the intended target region. If stem cells transiently express a viral fusogen, they might rapidly adhere and so be maintained long term in the heart. The added advantage of pH sensitive fusogens, such as VSV-G, is the ability to control activity such that cells only fuse at pH lower than 6.5. This has major implications for inducing temporally (the window during ischemia) and spatially (the ischemic region) regulated fusion* in vivo*. In fact, vMSCs delivered to the heart were found in the patch and in damaged myocardium fused with mouse cells. The ability for VSV-G to induce fusion in the patch may be due to close proximity to the ischemic region, causing the environment to be more acidic or by the remodeling of the collagen patch [48]. Collagen remodeling has been shown to occur via MSC secretion of matrix metalloproteinases (matrixins), serine proteases, and cysteine proteases [85]. While matrixins are active at neutral pH, serine and cysteine proteases are active at acidic pH, indirectly demonstrating cells are able to make the microenvironment acidic [86]. Taken together, the induction of cell fusion in the heart could exert functional benefit via multiple mechanisms.

A primary limitation of this approach is introducing viral machinery to an already damaged recipient. The entire virion, VSV, is known to be immunogenic and, at high enough concentrations, is lethal to mice [87]. Purified VSV-G or VSV-G reconstituted in lipid bilayers administered to *in vitro *cell culture is mitogenic (>0.8 *μ*g/mL) [88]. Interestingly, if the lipid concentrations were increased, while VSV-G concentration was held constant, the mitogenicity decreased, suggesting that the spacing of VSV-G in the membrane plays a role. Confirming the importance of VSV-G arrangement, Ochsenbein et al. demonstrated that 1,000 times more antibody is produced by C57BL/6 mice against highly organized VSV-G on the nucleocapsid of intact VSV versus poorly organized VSV-G in micelles [89]. The amount of viral proteins we delivered (based on the mass of the protein [39], the proteins expressed per cell combined with the number of cells delivered) is 7 orders of magnitude below the reported amount to elicit an immune response [88] and we express only the fusogen and not the entire virion. Even if methods were developed to increase expression levels per cell and/or in combination with high numbers of cells, spacing could be evaluated to avoid immune responses. However, based on the reported concentration required to elicit a response, delivery of vMSCs as prepared in this study would not trigger a response.

While vMSCs may not be immunogenic, transfection itself may cause adverse genetic effects. For instance, stable transfection with most viral systems causes integration of the gene at a random site in the genome [90–92]. When mutagenesis occurs, integration may occur at a site that interferes with cells ability to regulate itself, resulting in deregulation of proliferation and tumorigenesis [93, 94]. In addition to experimental evidence of malignancy, this has been seen clinically ([95, 96], reviewed in [97]). Here, transfection is largely transient and only rarely integrates into the genome. Clinical use would require further safeguards, perhaps including liposomal delivery of the protein.

## 5. Conclusion

The data presented support the utility of VSV-G-mediated fusion to study the effects of stem cell fusion on cell reprogramming and functional improvement of tissues including the heart. Future studies may also employ VSV-G to rescue damaged cells of other ischemic tissues in the body, or even selectively target cells for destruction. For example, the microenvironment of tumors and the overactive osteoclasts in Paget's disease are below the pH threshold necessary to activate the conformational change in VSV-G. Local administration of VSV-G in liposomes containing toxic factors or highly acidic pH to this microenvironment may fuse with these poorly regulated cells and dampen their detrimental effect.

## Supplementary Material

The supplementary information contains representative images of MSC-CM fusion products and corresponding fusion partners (MSCs and CMs) following immunocytochemistry for expression of CD73, CD90, CD105 and sarcomeric myosin proteins. Images show that MSC-CM fusion products can contain two or more nuclei or a single (sometimes enlarged) nucleus.
Supplementary Figure 1. Characterization of phenotype of human mesenchymal stem cells and HL-1 cardiomyocytes and vMSC-CM fusion products. A) MSC and CM populations used for these studies expressed CD73 (green) and CD90 (green), while only MSCs expressed CD105 (green) and only CMs expressed MF20 (red). Shown are representative images of cells following immunofluorescence labeling for each respective marker (+) or secondary antibody only (-) and counterstained with DAPI (blue). Scale bar = 50 **μ**m. B) Representative CD105+/MF20+ cells (white arrows) following pH-induced fusion of vMSC and CM. Scale bar = 50 **μ**m.

## References

[1] Tomita S, Li RK, Weisel RD (1999). Autologous transplantation of bone marrow cells improves damaged heart function. *Circulation*.

[2] Amado LC, Saliaris AP, Schuleri KH (2005). Cardiac repair with intramyocardial injection of allogeneic mesenchymal stem cells after myocardial infarction. *Proceedings of the National Academy of Sciences of the United States of America*.

[3] Chen SL, Fang WW, Ye F (2004). Effect on left ventricular function of intracoronary transplantation of autologous bone marrow mesenchymal stem cell in patients with acute myocardial infarction. *American Journal of Cardiology*.

[4] Ma J, Ge J, Zhang S (2005). Time course of myocardial stromal cell-derived factor 1 expression and beneficial effects of intravenously administered bone marrow stem cells in rats with experimental myocardial infarction. *Basic Research in Cardiology*.

[5] Shake JG, Gruber PJ, Baumgartner WA (2002). Mesenchymal stem cell implantation in a swine myocardial infarct model: engraftment and functional effects. *Annals of Thoracic Surgery*.

[6] Mouiseddine M, François S, Semont A (2007). Human mesenchymal stem cells home specifically to radiation-injured tissues in a non-obese diabetes/severe combined immunodeficiency mouse model. *British Journal of Radiology*.

[7] Nagaya N, Fujii T, Iwase T (2004). Intravenous administration of mesenchymal stem cells improves cardiac function in rats with acute myocardial infarction through angiogenesis and myogenesis. *American Journal of Physiology*.

[8] Chen G, Nayan M, Duong M (2010). Marrow stromal cells for cell-based therapy: the role of antiinflammatory cytokines in cellular cardiomyoplasty. *Annals of Thoracic Surgery*.

[9] Kamihata H, Matsubara H, Nishiue T (2001). Implantation of bone marrow mononuclear cells into ischemic myocardium enhances collateral perfusion and regional function via side supply of angioblasts, angiogenic ligands, and cytokines. *Circulation*.

[10] Kinnaird T, Stabile E, Burnett MS (2004). Marrow-derived stromal cells express genes encoding a broad spectrum of arteriogenic cytokines and promote *in vitro* and *in vivo* arteriogenesis through paracrine mechanisms. *Circulation Research*.

[11] Kawada H, Fujita J, Kinjo K (2004). Nonhematopoietic mesenchymal stem cells can be mobilized and differentiate into cardiomyocytes after myocardial infarction. *Blood*.

[12] Deb A, Wang S, Skelding KA, Miller D, Simper D, Caplice NM (2003). Bone marrow-derived cardiomyocytes are present in adult human heart: a study of gender-mismatched bone marrow transplantation patients. *Circulation*.

[13] Nygren JM, Jovinge S, Breitbach M (2004). Bone marrow-derived hematopoietic cells generate cardiomyocytes at a low frequency through cell fusion, but not transdifferentiation. *Nature Medicine*.

[14] Lin HP, Vincenz C, Eliceiri KW, Kerppola TK, Ogle BM (2010). Bimolecular fluorescence complementation analysis of eukaryotic fusion products. *Biology of the Cell*.

[15] Matsuura K, Wada H, Nagai T (2004). Cardiomyocytes fuse with surrounding noncardiomyocytes and reenter the cell cycle. *Journal of Cell Biology*.

[16] Oh H, Bradfute SB, Gallardo TD (2003). Cardiac progenitor cells from adult myocardium: homing, differentiation, and fusion after infarction. *Proceedings of the National Academy of Sciences of the United States of America*.

[17] Zhang S, Wang D, Estrov Z (2004). Both cell fusion and transdifferentiation account for the transformation of human peripheral blood CD34-positive cells into cardiomyocytes *in vivo*. *Circulation*.

[18] Waddington CH (1957). *The Strategy of Genes*.

[19] Briggs R, King TJ (1952). Transplantation of living nuclei from blastula cells into enucleated frogs' eggs. *Proceedings of the National Academy of Sciences of the United States of America*.

[20] Wilmut I, Schnieke AE, McWhir J, Kind AJ, Campbell KHS (1997). Viable offspring derived from fetal and adult mammalian cells. *Nature*.

[21] Matveeva NM, Shilov AG, Kaftanovskaya EM (1998). *In vitro* and *in vivo* study of pluripotency in intraspecific hybrid cells obtained by fusion of murine embryonic stem cells with splenocytes. *Molecular Reproduction and Development*.

[22] Yu J, Vodyanik MA, Smuga-Otto K (2007). Induced pluripotent stem cell lines derived from human somatic cells. *Science*.

[23] Takahashi K, Tanabe K, Ohnuki M (2007). Induction of pluripotent stem cells from adult human fibroblasts by defined factors. *Cell*.

[24] Maherali N, Sridharan R, Xie W (2007). Directly reprogrammed fibroblasts showglobalepigeneticremodeling andwidespreadtissuecontribution. *Cell Stem Cell*.

[25] Okita K, Ichisaka T, Yamanaka S (2007). Generation of germline-competent induced pluripotent stem cells. *Nature*.

[26] Wernig M, Meissner A, Foreman R (2007). *In vitro* reprogramming of fibroblasts into a pluripotent ES-cell-like state. *Nature*.

[27] Huppertz B, Bartz C, Kokozidou M (2006). Trophoblast fusion: fusogenic proteins, syncytins and ADAMs, and other prerequisites for syncytial fusion. *Micron*.

[28] Oren-Suissa M, Podbilewicz B (2007). Cell fusion during development. *Trends in Cell Biology*.

[29] Zimmermann U, Vienken J (1982). Electric field-induced cell-to-cell fusion. *Journal of Membrane Biology*.

[30] Schneckenburger H, Hendinger A, Sailer R (2000). Cell viability in optical tweezers: high power red laser diode versus Nd:YAG laser. *Journal of Biomedical Optics*.

[31] Steubing RW, Cheng S, Wright WH, Numajiri Y, Berns MW (1991). Laser induced cell fusion in combination with optical tweezers: the laser cell fusion trap. *Cytometry*.

[32] Bartal AH, Hirshaut Y (1987). *Methods of Hybridoma Formation*.

[33] Radomska HS, Eckhardt LA (1995). Mammalian cell fusion in an electroporation device. *Journal of Immunological Methods*.

[34] Lentz BR, Lee J (1999). Poly(ethylene glycol) (PEG)-mediated fusion between pure lipid bilayers: a mechanism in common with viral fusion and secretory vesicle release?. *Molecular Membrane Biology*.

[35] Eckert DM, Kim PS (2001). Mechanisms of viral membrane fusion and its inhibition. *Annual Review of Biochemistry*.

[36] Sapir A, Avinoam O, Podbilewicz B, Chernomordik LV (2008). Viral and developmental cell fusion mechanisms: conservation and divergence. *Developmental Cell*.

[37] Chen EH, Olson EN (2005). Unveiling the mechanisms of cell-cell fusion. *Science*.

[38] Jeetendra E, Robison CS, Albritton LM, Whitt MA (2002). The membrane-proximal domain of vesicular stomatitis virus G protein functions as a membrane fusion potentiator and can induce hemifusion. *Journal of Virology*.

[39] Sun X, Belouzard S, Whittaker GR (2008). Molecular architecture of the bipartite fusion loops of vesicular stomatitis virus glycoprotein G, a class III viral fusion protein. *Journal of Biological Chemistry*.

[40] Dunning J, Hunter S, Kendall SWH, Wallis J, Owens WA (2006). Coronary bypass grafting using crossclamp fibrillation does not result in reliable reperfusion of the myocardium when the crossclamp is intermittently released: a prospective cohort study. *Journal of Cardiothoracic Surgery*.

[41] Boumans MLL, Diris JHC, Nap M (2001). Creatine kinase isoenzyme MB (CKMB) controversy: perimortal tissue acidosis may explain the absence of CKMB in myocardium at autopsy. *Clinical Chemistry*.

[42] Lange R, Kloner RA, Zierler M (1983). Time course of ischemic alterations during normothermic and hypothermic arrest and its reflection by on-line monitoring of tissue pH. *Journal of Thoracic and Cardiovascular Surgery*.

[43] Trivedi P, Hematti P (2008). Derivation and immunological characterization of mesenchymal stromal cells from human embryonic stem cells. *Experimental Hematology*.

[44] Claycomb WC, Lanson NA, Stallworth BS (1998). HL-1 cells: a cardiac muscle cell line that contracts and retains phenotypic characteristics of the adult cardiomyocyte. *Proceedings of the National Academy of Sciences of the United States of America*.

[45] Takada A, Robison C, Goto H (1997). A system for functional analysis of Ebola virus glycoprotein. *Proceedings of the National Academy of Sciences of the United States of America*.

[46] Fredericksen BL, Whitt MA (1995). Vesicular stomatitis virus glycoprotein mutations that affect membrane fusion activity and abolish virus infectivity. *Journal of Virology*.

[47] Michael LH, Entman ML, Hartley CJ (1995). Myocardial ischemia and reperfusion: a murine model. *American Journal of Physiology*.

[48] Kouris NA, Squirrell JM, Jung JP (2011). A nondenatured, noncrosslinked collagen matrix to deliver stem cells to the heart. *Regenerative Medicine*.

[49] Ogle BM, Butters KA, Plummer TB (2004). Spontaneous fusion of cells between species yields transdifferentiation and retroviral transfer *in vivo*.. *The FASEB Journal*.

[50] Snider MD, Robbins PW (1982). Transmembrane organization of protein glycosylation. Mature oligosaccharide-lipid is located on the luminal side of microsomes from Chinese hamster ovary cells. *Journal of Biological Chemistry*.

[51] Simmons G, Gosalia DN, Rennekamp AJ, Reeves JD, Diamond SL, Bates P (2005). Inhibitors of cathepsin L prevent severe acute respiratory syndrome coronavirus entry. *Proceedings of the National Academy of Sciences of the United States of America*.

[52] Katz FN, Rothman JE, Lingappa VR (1977). Membrane assembly *in vitro*: synthesis, glycosylation, and asymmetric insertion of a transmembrane protein. *Proceedings of the National Academy of Sciences of the United States of America*.

[53] Bajpai R, Lesperance J, Kim M, Terskikh AV (2008). Efficient propagation of single cells accutase-dissociated human embryonic stem cells. *Molecular Reproduction and Development*.

[54] Carneiro FA, Ferradosa AS, Da Poian AT (2001). Low pH-induced conformational changes in vesicular stomatitis virus glycoprotein involve dramatic structure reorganization. *Journal of Biological Chemistry*.

[55] Clague MJ, Schoch C, Zech L, Blumenthal R (1990). Gating kinetics of pH-activated membrane fusion of vesicular stomatitis virus with cells: stopped-flow measurements by dequenching of octadecylrhodamine fluorescence. *Biochemistry*.

[56] Chen CS, Mrksich M, Huang S, Whitesides GM, Ingber DE (1997). Geometric control of cell life and death. *Science*.

[57] Oh S, Brammer KS, Li YSJ (2009). Stem cell fate dictated solely by altered nanotube dimension. *Proceedings of the National Academy of Sciences of the United States of America*.

[58] McBeath R, Pirone DM, Nelson CM, Bhadriraju K, Chen CS (2004). Cell shape, cytoskeletal tension, and RhoA regulate stem cell lineage commitment. *Developmental Cell*.

[59] Laflamme MA, Chen KY, Naumova AV (2007). Cardiomyocytes derived from human embryonic stem cells in pro-survival factors enhance function of infarcted rat hearts. *Nature Biotechnology*.

[60] Alvarez-Dolado M, Pardal R, Garcia-Verdugo JM (2003). Fusion of bone-marrow-derived cells with Purkinje neurons, cardiomyocytes and hepatocytes. *Nature*.

[61] Noiseux N, Gnecchi M, Lopez-Ilasaca M (2006). Mesenchymal stem cells overexpressing Akt dramatically repair infarcted myocardium and improve cardiac function despite infrequent cellular fusion or differentiation. *Molecular Therapy*.

[62] Xu W, Zhang X, Qian H (2004). Mesenchymal stem cells from adult human bone marrow differentiate into a cardiomyocyte phenotype *in vitro*. *Experimental Biology and Medicine*.

[63] Balana B, Nicoletti C, Zahanich I (2006). 5-azacytidine induces changes in electrophysiological properties of human mesenchymal stem cells. *Cell Research*.

[64] Wakitani S, Saito T, Caplan AI (1995). Myogenic cells derived from rat bone marrow mesenchymal stem cells exposed to 5-azacytidine. *Muscle and Nerve*.

[65] Santiago JA, Pogemiller R, Ogle BM (2009). Heterogeneous differentiation of human mesenchymal stem cells in response to extended culture in extracellular matrices. *Tissue Engineering A*.

[66] Xu M, Wani M, Dai YS (2004). Differentiation of bone marrow stromal cells into the cardiac phenotype requires intercellular communication with myocytes. *Circulation*.

[67] Metzele R, Alt C, Bai X (2011). Human adipose tissue-derived stem cells exhibit proliferation potential and spontaneous rhythmic contraction after fusion with neonatal rat cardiomyocytes. *The FASEB Journal*.

[68] Zhang Y, Chan DC (2007). New insights into mitochondrial fusion. *FEBS Letters*.

[69] Acquistapace A, Bru T, Lesault PF (2011). Human mesenchymal stem cells reprogram adult cardiomyocytes toward a progenitor-like state through partial cell fusion and mitochondria transfer. *Stem Cells*.

[70] Terada N, Hamazaki T, Oka M (2002). Bone marrow cells adopt the phenotype of other cells by spontaneous cell fusion. *Nature*.

[71] Pomerantz J, Blau HM (2004). Nuclear reprogramming: a key to stem cell function in regenerative medicine. *Nature Cell Biology*.

[72] Anversa P, Leri A, Kajstura J (2006). Cardiac Regeneration. *Journal of the American College of Cardiology*.

[73] Reinecke H, Minami E, Poppa V, Murry CE (2004). Evidence for fusion between cardiac and skeletal muscle cells.. *Circulation Research*.

[74] Taylor DA, Atkins BZ, Hungspreugs P (1998). Regenerating functional myocardium: improved performance after skeletal myoblast transplantation. *Nature Medicine*.

[75] Hagège AA, Carrion C, Menasché P (2003). Viability and differentiation of autologous skeletal myoblast grafts in ischaemic cardiomyopathy. *The Lancet*.

[76] Orlic D, Kajstura J, Chimenti S (2001). Bone marrow cells regenerate infarcted myocardium. *Nature*.

[77] Singla DK, Hacker TA, Ma L (2006). Transplantation of embryonic stem cells into the infarcted mouse heart: formation of multiple cell types. *Journal of Molecular and Cellular Cardiology*.

[78] Zhang J, Wilson GF, Soerens AG (2009). Functional cardiomyocytes derived from human induced pluripotent stem cells. *Circulation Research*.

[79] Ieda M, Fu JD, Delgado-Olguin P (2010). Direct reprogramming of fibroblasts into functional cardiomyocytes by defined factors. *Cell*.

[80] Zhang M, Methot D, Poppa V, Fujio Y, Walsh K, Murry CE (2001). Cardiomyocyte grafting for cardiac repair: graft cell death and anti-death strategies. *Journal of Molecular and Cellular Cardiology*.

[81] Hou D, Youssef EAS, Brinton TJ (2005). Radiolabeled cell distribution after intramyocardial, intracoronary, and interstitial retrograde coronary venous delivery: implications for current clinical trials. *Circulation*.

[82] Freyman T, Polin G, Osman H (2006). A quantitative, randomized study evaluating three methods of mesenchymal stem cell delivery following myocardial infarction. *European Heart Journal*.

[83] Bursac N, Loo Y, Leong K, Tung L (2007). Novel anisotropic engineered cardiac tissues: studies of electrical propagation. *Biochemical and Biophysical Research Communications*.

[84] Kochupura PV, Azeloglu EU, Kelly DJ (2005). Tissue-engineered myocardial patch derived from extracellular matrix provides regional mechanical function. *Circulation*.

[85] Mauney J, Olsen BR, Volloch V (2010). Matrix remodeling as stem cell recruitment event: a novel *in vitro* model for homing of human bone marrow stromal cells to the site of injury shows crucial role of extracellular collagen matrix. *Matrix Biology*.

[86] Etherington DJ (1977). Collagen degradation. *Annals of the Rheumatic Diseases*.

[87] Flanagan EB, Ball LA, Wertz GW (2000). Moving the glycoprotein gene of vesicular stomatitis virus to promoter-proximal positions accelerates and enhances the protective immune response. *Journal of Virology*.

[88] Goodman-Snitkoff G, Mannino RJ, McSharry JJ (1981). The glycoprotein isolated from vesicular stomatitis virus is mitogenic for mouse B lymphocytes. *Journal of Experimental Medicine*.

[89] Ochsenbein AF, Pinschewer DD, Odermatt B, Ciurea A, Hengartner H, Zinkernagel RM (2000). Correlation of T cell independence of antibody responses with antigen dose reaching secondary lymphoid organs: implications for splenectomized patients and vaccine design. *Journal of Immunology*.

[90] Stocking C, Bergholz U, Friel J (1993). Distinct classes of factor-independent mutants can be isolated after retroviral mutagenesis of a human myeloid stem cell line. *Growth Factors*.

[91] Kustikova OS, Wahlers A, Kühlcke K (2003). Dose finding with retroviral vectors: correlation of retroviral vector copy numbers in single cells with gene transfer efficiency in a cell population. *Blood*.

[92] Shih CC, Stoye JP, Coffin JM (1988). Highly preferred targets for retrovirus integration. *Cell*.

[93] Li Z, Düllmann J, Schiedlmeier B (2002). Murine leukemia induced by retroviral gene marking. *Science*.

[94] Hacein-Bey-Abina S, Von Kalle C, Schmidt M (2003). LMO2-Associated Clonal T Cell Proliferation in Two Patients after Gene Therapy for SCID-X1. *Science*.

[95] Hacein-Bey-Abina S, Von Kalle C, Schmidt M (2003). A serious adverse event after successful gene therapy for X-linked severe combined immunodeficiency. *The New England Journal of Medicine*.

[96] Howe SJ, Mansour MR, Schwarzwaelder K (2008). Insertional mutagenesis combined with acquired somatic mutations causes leukemogenesis following gene therapy of SCID-X1 patients. *Journal of Clinical Investigation*.

[97] Bushman FD (2007). Retroviral integration and human gene therapy. *Journal of Clinical Investigation*.

