# Supplementary figures and images for: Directed Fusion of Mesenchymal Stem Cells with Cardiomyocytes via VSV-G Facilitates Stem Cell Programming
Source: Stem Cells Int. 2012 May 30;2012:414038. doi: 10.1155/2012/414038 (PMC3369562; doi:10.1155/2012/414038)

A

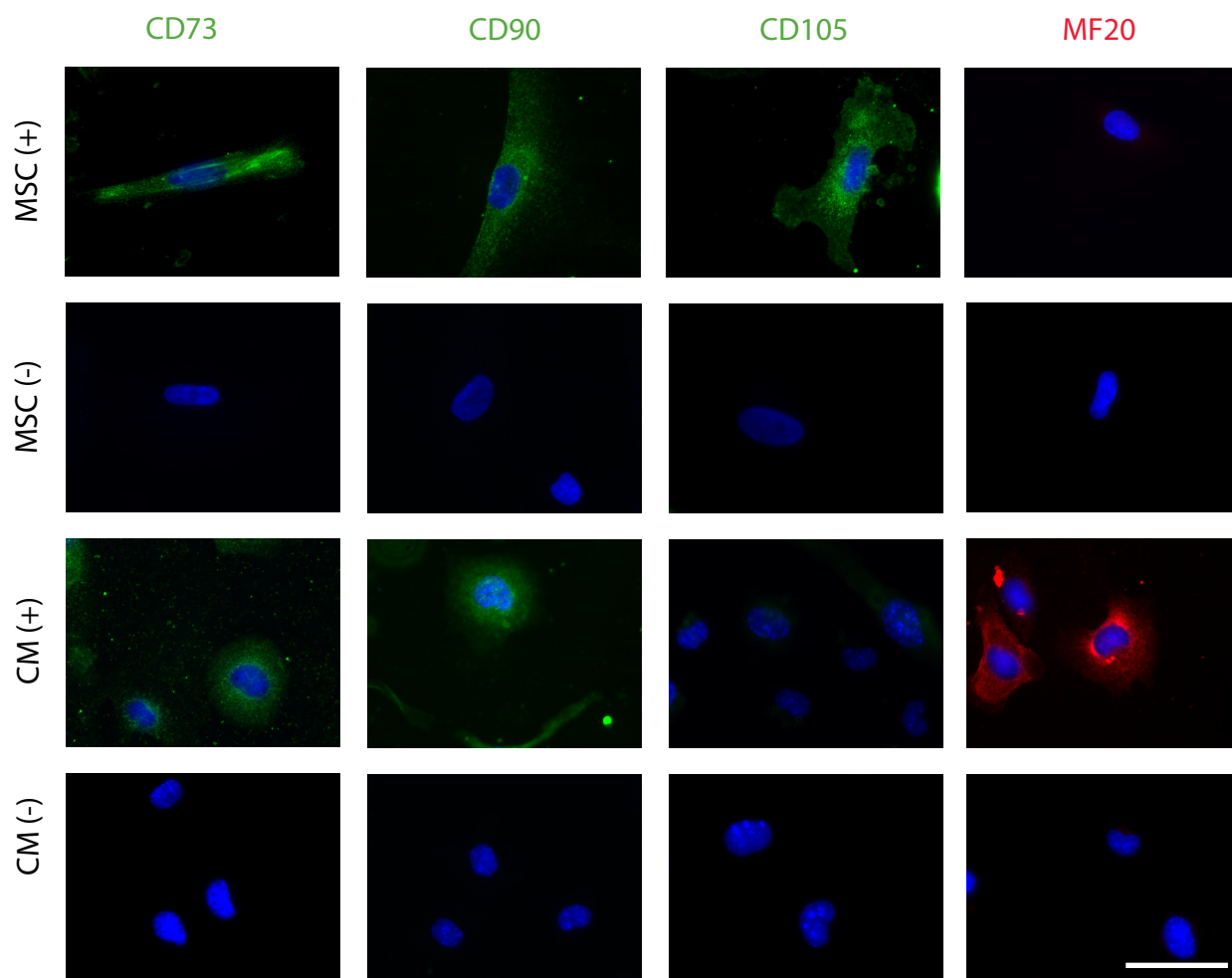

B

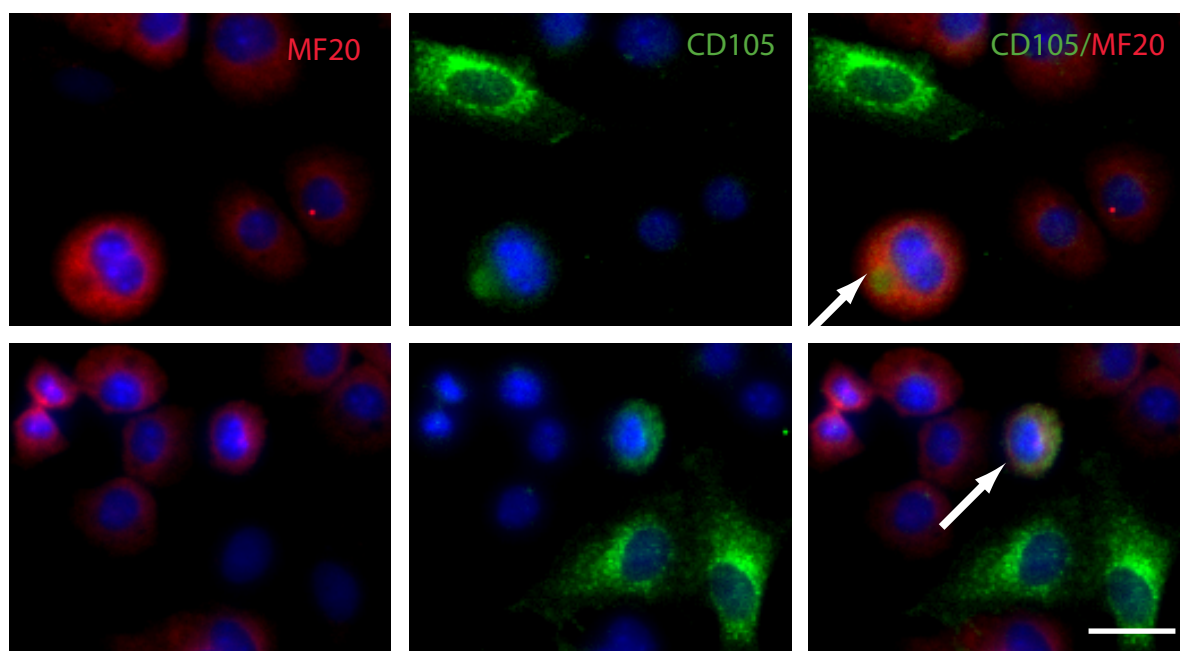

Supplement: Supplementary file 1 — The supplementary information contains representative images of MSC-CM fusion products and corresponding fusion partners (MSCs and CMs) following immunocytochemistry for expression of CD73, CD90, CD105 and sarcomeric myosin proteins. Images show that MSC-CM fusion products can contain two or more nuclei or a single (sometimes enlarged) nucleus. Supplementary Figure 1. Characterization of phenotype of human mesenchymal stem cells and HL-1 cardiomyocytes and vMSC-CM fusion products. A) MSC and CM populations used for these studies expressed CD73 (green) and CD90 (green), while only MSCs expressed CD105 (green) and only CMs expressed MF20 (red). Shown are representative images of cells following immunofluorescence labeling for each respective marker (+) or secondary antibody only (-) and counterstained with DAPI (blue). Scale bar = 50 μm. B) Representative CD105+/MF20+ cells (white arrows) following pH-induced fusion of vMSC and CM. Scale bar = 50 μm. [file 414038.f1.pdf]
